# The Influence of the COVID-19 Pandemic on Traumatic Spinal Cord Injury: A Six-Year Population-Based Retrospective Analysis

**DOI:** 10.21203/rs.3.rs-9304752/v1

**Published:** 2026-05-11

**Authors:** Kelsey Potter-Baker, Blake Martin, Maci Oestreich, Daniel Salinas, Rex Marco, Ann Van de Winckel

**Affiliations:** University of Texas Rio Grande Valley School of Medicine; usa

## Abstract

**Study Design:**

Retrospective observational population-based study

**Objective:**

Evaluate traumatic spinal cord injury (tSCI) epidemiology before, during, and after the coronavirus disease 2019 (COVID-19) pandemic in Texas

**Setting:**

Texas Department of State Health Services Trauma Database

**Methods:**

The trauma database was queried for ICD-10 diagnosis codes pertinent to tSCI (S14, S24, S34, T093, and T91.3) from 2019 to 2024. Outcome measures included tSCI diagnosis, cause of injury, region of tSCI, age, sex, ethnicity, and public health region. A generalized estimating equations (GEE) model was utilized with a binomial distribution to evaluate predictors of injury status (tSCI vs. non-tSCI trauma). Data was evaluated in the pre-pandemic (2019), pandemic (2020-21) and post-pandemic (2022-24) period.

**Results:**

A total of 7,901 tSCIs occurred over the study period. Compared with the pandemic period, the pre-pandemic year demonstrated a rate of 1,153 cases per year (OR = 1.96). This rate declined substantially during the pandemic (2020–2021) to 468.5 cases per year, followed by a marked rebound in the post-pandemic period (2022–2024) to 1,937 cases per year (OR = 3.08). Age, sex, ethnicity, injury mechanism, and Public Health Region were all independently associated with tSCI risk in multivariable GEE models.

**Conclusion:**

SCI incidence declined during the COVID-19 pandemic, likely due to reduced exposure and healthcare avoidance, and subsequently rebounded markedly in the post-pandemic period, with disproportionate increases observed in Black and Asian groups and overall lower risk in non-PHR 3 regions. These changes in tSCI epidemiology provide critical insight into future research and healthcare priorities.

## Introduction

Spinal cord injuries (SCIs) are complex medical conditions that often result from traumatic events such as motor vehicle collisions and falls. In the United States, approximately 300,000 individuals are currently living with SCI, with an estimated 18,000 new cases occurring each year (1). However, emerging evidence suggests that the epidemiology of SCI may have shifted following the onset of the coronavirus disease 2019 (COVID-19) pandemic (2).

Prior work suggests that changes in physical and social activity during the COVID-19 pandemic altered exposure to etiologies associated with SCI (3). For example, the lowest incidence of traumatic SCI (tSCI) was observed during periods of strict lockdown—particularly among individuals over 45 years of age and males—alongside a shift in etiology, including increased cases related to self-harm due to change in mental health (4). Beyond these epidemiological changes, the pandemic also adversely affected healthcare utilization, access, and outcomes for individuals with tSCI, with multiple studies reporting increased mortality rates during this period (3, 5-7). While individuals with pre-existing SCI appeared to remain relatively stable clinically, they experienced significant challenges related to reduced social support (1).

International SCI societies have called for improved evidence on the impact of COVID-19 on SCI (8). Here, we seek to expand epidemiological knowledge of tSCI in a Texas population-based analysis. Our primary objective was to examine tSCI before, during, and after the COVID-19 pandemic, with particular emphasis on changes in incidence and injury location (cervical, thoracic, and lumbar). Within Texas, traumatic injuries, including SCI are mandatory reporting events to the state health department (9, 10). Thus, we evaluated population-level epidemiologic trends across a diverse region to better define the impact of COVID-19 on tSCI, with a focus on the post-pandemic period—an area that remains largely underexplored. We hypothesized that tSCI incidence would decline during the pandemic and subsequently increase in the post-pandemic period, and that mechanisms of injury would differ across these intervals. Overall, we sought to identify population-level patterns that may inform future treatment strategies, rehabilitation paradigms, and healthcare delivery for individuals with tSCI.

## Methods

### Study Design, Data Source and Regulatory Approval

We conducted a retrospective population-based analysis of tSCI in the State of Texas. Trauma injury data was obtained from the Trauma Registry from the Texas Department of State Health Services (DSHS) (11). We evaluated data from all reported trauma events between January 2018 to December 2024. This study was reviewed by the University of Texas Rio Grande Valley Institutional Review Board and was determined to be non-human subjects’ research. No additional informed consent was required.

### Data Preprocessing and Study Population Identification

Datasets from 2018 through 2024, comprising 1,107,240 cases were merged based on common variables. The obtained datasets were cleaned by merging related files and standardizing data fields where appropriate. To facilitate interpretation and statistical analysis, variables with unstandardized response options were consolidated into distinct categories based on the most relevant features. For example, causes of injury were categorized using ICD-10 code information. During data preprocessing, we observed that records from 2018 were substantially incomplete. Verification with the Texas Department of State Health Services (DSHS) indicated that reporting processes prior to 2019 were not fully standardized, which contributed to the missing data observed for 2018. Consequently, data from 2018 were excluded from the present analysis.

We identified cases with tSCI in the preprocessed dataset using injury diagnosis (ICD-10) classification codes. Specifically, cases were included in analysis that were coded with S14 (cervical SCI), S24 (thoracic SCI), S34 (lumbar/sacral SCI), T09.3 (unspecified SCI) or T91.3 (late effects of SCI) for cause of injury within the dataset. All duplicate diagnoses or classifications within cases or subjects were removed.

### Demographic variables and tSCI Etiology Characteristics

For all identified tSCI cases, demographic and injury etiology was extracted for analysis, including year of injury, age, sex, ethnicity, spinal cord injury region, and three-digit ZIP codes of the region of diagnosis. Age for each case was categorized into the following groups: children (under 12 years), teens (12–18 years), young adults (19–34 years), adults (35–49 years), middle-aged adults (50–64 years), seniors (65–79 years), and elderly (80 years and older). tSCI cases were categorized across three temporal periods: pre-pandemic (2019), pandemic (2020–2021), and post-pandemic (2022–2024). These periods were selected to align with timelines used in other regional studies of COVID-19–related healthcare and trauma trends, while recognizing that mitigation measures and restrictions varied across different regions of Texas (12).

Causes of injury (COI) were categorized into ten broad groups based on descriptive text, drawing from standardized injury epidemiology frameworks such as ICD-10 external cause codes and CDC matrices (13).

1. Transport Injuries (Unintentional): pedestrian transport injuries (e.g. trauma sustained on foot by transport vehicle), non-motorized transport injuries (e.g. trauma sustained by human-powered or animal transport), or motor vehicle traffic injuries
2. Exposure to Forces of Nature/Poisoning: exposure to forces of nature, poisoning by substances, or exposure to radiation
3. Assault/Homicide: assault by bodily force or object, intimate partner violence, homicide, terrorism/violent extremism, legal intervention, or war and civil conflict injuries
4. Intentional Self-Harm
5. Exposure to Inanimate Mechanical Forces: exposure to inanimate mechanical forces, struck by or against object, or constriction/crushing injuries
6. Complications of Medical Care/Asphyxiation: complications of medical and surgical care, infectious and parasitic diseases, or aspiration/choking
7. Exposure to Animate Mechanical Forces/Overexertion: contact with animals or plants, sports and leisure activity injuries, or overexertion and strenuous movements
8. Falls (Unintentional)
9. Fire/Burns and Firearms (by Intent): firearms, fire, or burns (unintentional)
10. Undetermined/Other: uncategorized or unspecified entries

Each tSCI case was also categorized into their associated Public Health Region (PHR) using the 3-digit zip code from the database. PHRs are administrative divisions used by health authorities to monitor population health and allocate resources. Data was categorized by PHR to allow for the assessment of regional disparities in injury mechanisms and healthcare access across the 11 designated health regions in Texas. PHR regions are summarized in Table 1.

### Data Analysis and Statistical Methods

Data management and statistical analyses were performed using R software (version 4.4.1, R Core Team, 2024). Descriptive statistics were used to summarize the distributions of demographic characteristics (sex, age group, ethnicity), injury etiology (cause of injury), year of injury, and geographic variation by Public Health Region (PHR).

#### Generalized Estimating Equations (GEE) Modeling.

To evaluate predictors of injury status (SCI vs. non-SCI trauma), a GEE model was utilized with a binomial distribution and logit link function, implemented via the geepack package in R. GEE was selected to account for within-subject correlation arising from repeated observations per individual as multiple SCI (e.g., cervical, thoracic, and lumbar). An independence working correlation structure was specified and robust (sandwich) standard errors were used to obtain population-averaged inference.

#### Model Specification and Reference Categories.

Statistical reference categories were primarily selected based on the highest frequency of occurrence within the dataset to ensure maximum statistical power and stability of the model estimates. Specifically, young adults and males were designated as reference groups due to their predominance in traumatic injury prevalence. For temporal and etiological analyses, the COVID-19 pandemic period and Transport Injuries were utilized as baseline comparators to facilitate the interpretation of shifts in injury patterns relative to historical norms and the leading cause of injury.

The final model included injury status as the dependent variable and spinal injury level as the primary predictor. To examine whether the association between spinal level and injury status differed across covariates, interaction terms were specified between spinal level and sex, age group, ethnicity group, category of injury onset (COI), PHR, and COVID-19 periods. All corresponding main effects were included in the model. Statistical significance was evaluated at α < 0.05, and results are reported as odds ratios with 95% confidence intervals.

#### Handling of Missing Data.

For GEE modeling, missing data were handled using complete-case analysis (listwise deletion). Across the full dataset (2019–2024), only observations with complete information for the outcome, the repeated-measures factor (spinal level), and all covariates were retained for model analysis. This resulted in the exclusion of 1,795 participants with tSCI (23% of all tSCI cases), yielding a final analytic tSCI sample of 6,106 cases (77%).

## Results

**Figure 1:**
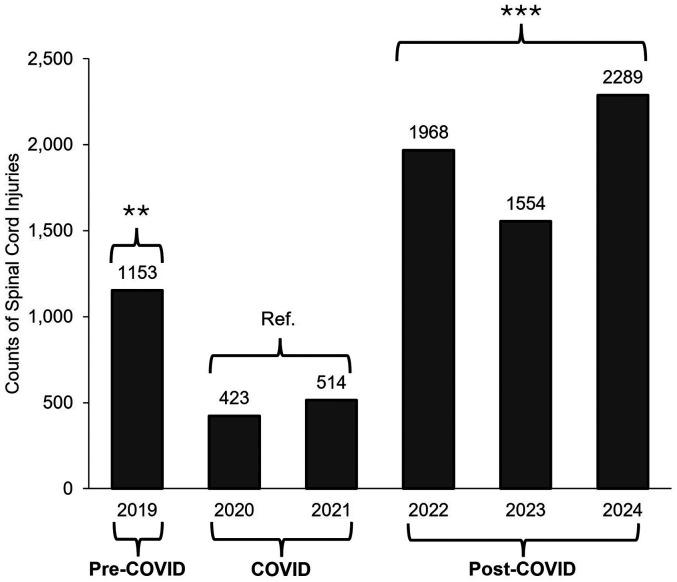
Annual counts of traumatic spinal cord injuries (tSCI) from 2019–2024 in Texas. Pre-COVID was defined as 2019, COVID as 2020 – 2021 and post-COVID as 2022 – 2024. Multivariate GEE model comparisons use COVID as the reference period (*p* < 0.01: **, *p* < 0.001: ***).

A total of 7,901 tSCIs occurred during the study period. The pre-COVID period accounted for 14.9% of all injuries (n = 1,153), the COVID period accounted for 29.9% (n = 937), and the post-COVID period accounted for the largest proportion at 55.2% (n = 5,811) (Fig. 1). Rates of injuries per year for pre-COVID, COVID and post-COVID were 1153, 468.5 and 1937 cases per year, respectively. Notably, rates of tSCI were doubled following the COVID period, with the highest rate occurring in 2024.

**Figure 2:**
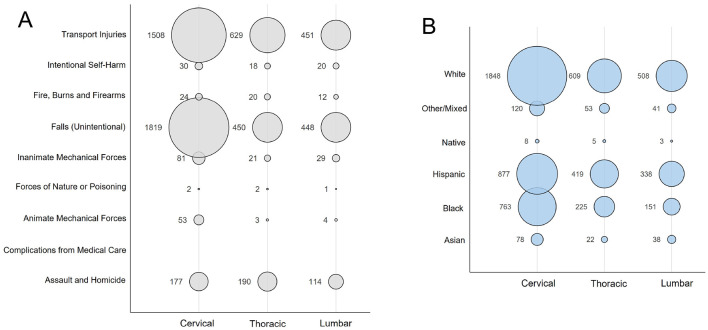
Frequency of traumatic spinal cord injury (tSCI) during the 6-year period at vertebral level based on cause of injury (COI) (A) and ethnicity (B). Bubble plot denotes the frequency of events during the six-year period. Size of circles is representative of the frequency count.

The 6,106 tSCI cases that were evaluated using a GEE model demonstrated a range of COI, with transport injuries and unintentional falls occurring most frequently (Fig. 2A). In addition, cervical injuries were most common among the population, followed by thoracic and lumbar respectively. Most of the assessed population was classified as White, with Hispanic and Black individuals among the next most common (Fig. 2B).

**Figure 3:**
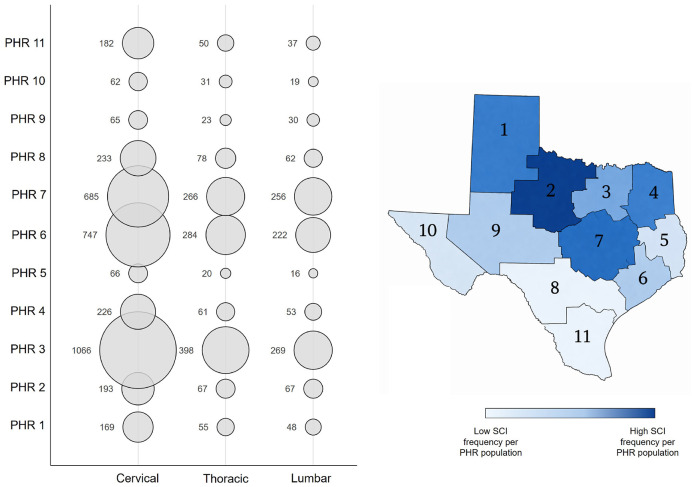
Frequency of traumatic spinal cord injury (tSCI) in Public Health Regions (PHR) in the state of Texas. Most tSCI events occurred in areas of highest population (PHR 3, 6 and 7), with cervical injuries the most common in every PHR. The total number of tSCI during the six-year period was evaluated in each PHR region as a function of the estimated 2021 population (Table 1). The highest SCI frequency per PHR population was observed in PHR 2 (Abilene area), with PHR 1 (Lubbock), PHR 4 (Tyler) and PHR 7 (Austin) also demonstrating higher frequency per population rates than the rest of the state. PHR region map was modified from the Texas Department of State Health Services (DSHS).

Included cases were spread across Texas, with cervical injuries remaining the most common for each PHR region (Fig. 3). We evaluated the frequency (counts) of SCI per PHR population (Table 1) and identified that the highest frequency of SCI per population occurred in PHR 2, with PHR 1, 4 and 7 also demonstrating higher frequency rates.

**Figure 4:**
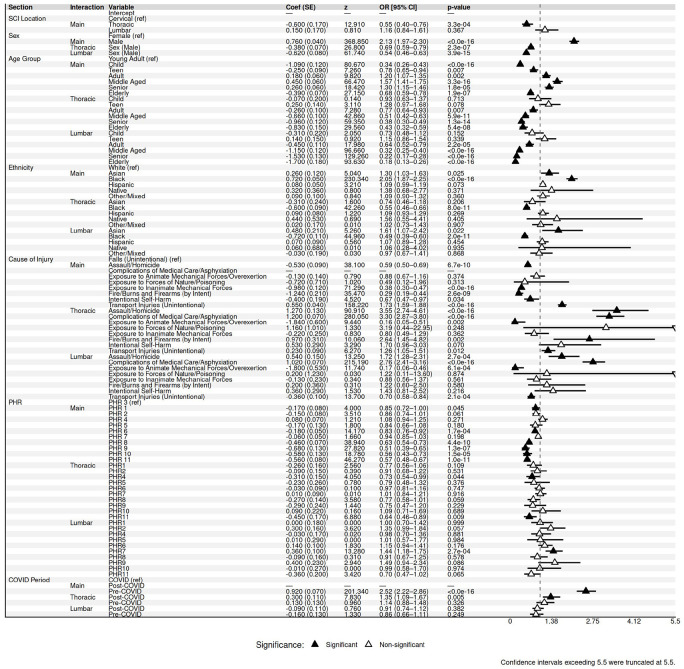
GEE Model Prediction of traumatic spinal cord injury (tSCI) versus non-SCI trauma. Shown are main effects and spinal-level interaction terms, reported as odds ratios with 95% confidence intervals, coefficients (SE), z-values, and *p*-values. The forest plot visualizes odds ratios for all predictors. Confidence intervals exceeding 5.5 were truncated for visualization. Reference categories were males, young adults, falls (unintentional), and COVID period

The GEE models, accounting for repeated observations, were statistically significant. tSCI occurrence was independently associated with pandemic period, sex, age, race/ethnicity, injury mechanism, public health region (PHR), and vertebral level of injury, relative to the reference categories (Fig. 4). The multivariate GEE model was conducted using an independent correlation structure across 103,482 clusters (maximum cluster size of 3), yielding an estimated scale parameter of 0.924.

### Age and Sex.

After adjusting for patient-level and regional factors, age and sex showed significant associations with injury location. Compared to the reference group (Young Adults), Teens showed a 22% lower risk of spinal injuries (OR 0.78, 95% CI 0.65–0.94, p = 0.007). A significant association was observed for sex; males comprised approximately 70% of the tSCI cases and had a 113% higher risk overall than females (OR 2.13, 95% CI 1.97–2.30, p < 0.0001). However, a significant interaction was observed for specific locations; males had a 46% lower risk than females of sustaining lumbar injuries (OR 0.54, 95% CI 0.46–0.63, p = 3.9e-15). Collectively, children and older adults consistently exhibited lower risk compared with young adults, whereas males experienced persistently higher risk across the study period (Fig. 4).

### Race and Ethnicity.

Black individuals experienced a 105% higher overall risk (OR 2.05, 95% CI 1.87–2.25, p < 0.0001), though this risk was lower for thoracic and lumbar injuries, indicating that cervical injuries were most common in Black tSCI. Asian individuals also showed a significant 30% higher risk overall (OR 1.30, 95% CI 1.03–1.63, p = 0.025) and a unique 61% higher risk specifically for lumbar injuries (OR 1.61, 95% CI 1.07–2.42, p = 0.022). In contrast, injury risk for Hispanic individuals did not differ significantly from White individuals (OR 1.09, 95% CI 0.99–1.19, p = 0.073).

### Injury Mechanisms.

Injury risk varied significantly by mechanism relative to unintentional falls. Overall, transport-related injuries were associated with a 73% higher overall risk (OR 1.73, 95% CI 1.59–1.88, p < 0.0001) and intentional self-harm with a 33% lower overall risk (OR 0.67, 95% CI 0.47–0.97, p = 0.034). Significant interactions were observed for specific vertebral locations: Assault/Homicide increased the risk of thoracic injuries by 255% (OR 3.55, 95% CI 2.74–4.61, p < 0.0001) and lumbar injuries by 72% (OR 1.72, 95% CI 1.28–2.31, p = 2.7e-04). Similarly, complications of medical care increased thoracic risk by 230% (OR 3.30, 95% CI 2.87–3.80, p < 0.0001) and lumbar risk by 176% (OR 2.76, 95% CI 2.41–3.16, p < 0.0001). Conversely, inanimate mechanical forces showed a 49% lower risk specifically for thoracic injuries (OR 0.51, 95% CI 0.39–0.65, p = 1.3e-07).

Collectively, thoracic tSCIs were more strongly associated with assault/homicide, complications to medical care or asphyxiation and fire-, burn-, and firearm-related mechanisms in the present dataset, consistent with their link to high-energy trauma. By contrast, compared to cervical SCIs, lumbar SCIs were significantly less likely to result from transport injuries, while thoracic injuries were more likely.

### Temporal Trends.

The temporal analysis revealed significant variation relative to the pandemic period. Compared to the COVID-19 reference period, the pre-COVID period saw a 152% higher risk of injury (OR 2.52, 95% CI 2.22–2.86, p < 0.0001). In the post-COVID period, there was a significant 35% increase in the risk of thoracic injuries (OR 1.35, 95% CI 1.09–1.67, p = 0.005).

### Regional Variation.

Regionally, nearly all Public Health Regions exhibited a lower overall risk compared to PHR 3 (Dallas-Fort Worth metropolitan area). PHR 11 (South Texas and Rio Grande Valley area) was associated with a 43% lower overall risk (OR 0.57, 95% CI 0.48–0.67, p = 1.0e-11), while PHR 8 (San Antonio area) demonstrated a 37% lower overall risk (OR 0.63, 95% CI 0.54–0.73, p = 4.4e-10). While some regional interactions suggested lower risk for specific vertebral levels (e.g., PHR 11 thoracic risk OR 0.70), these did not consistently reach the threshold for statistical significance.

## Discussion

Epidemiologic trends in tSCI following the COVID-19 pandemic may shape future priorities for research funding, healthcare resource allocation, and public health interventions. In this population-based retrospective analysis, we evaluated changes in patient demographics, injury location, and mechanisms of injury before, during, and after the pandemic. Overall, we found that age, injury location, ethnicity, and mechanism of injury were significant factors influencing the odds of tSCI during the study period.

### Role of COVID-19 Pandemic

We observed that tSCI declined substantially during the COVID-19 pandemic (Fig. 1), followed by a pronounced post-pandemic rebound that exceeded pre-pandemic levels. Adjusted analyses confirmed that both pre- and post-COVID periods were associated with significantly higher odds of tSCI compared with the pandemic period (Fig. 4). The sharp decline during the pandemic mirrored international findings and was likely driven by patient hesitancy to seek in-person care, overwhelmed healthcare systems, and reduced exposure to high-risk environments (15). For instance, multiple studies reported a correlation between reduced vehicular traffic, resulting from lockdown mandates, and lower SCI rates during the early pandemic (16-18). Although the reason for the post-pandemic increase remains uncertain, one potential explanation is that the lifting of lockdown restrictions was associated with increased mobility and participation in outdoor and high-energy activities, which may have elevated the risk of traumatic injury. Overall, our findings highlight the need for an adaptable healthcare system that can sustain trauma care during crises and support a gradual recovery as normal activity resumes. Further, it suggests that additional public health interventions may be needed to address the large post-pandemic rebound.

### Cause of Injury

In contrast to previous studies (19), we observed that transport-related injuries emerged as the strongest mechanism associated with SCI in adjusted analyses (Fig. 2A), consistently exceeding the risk associated with falls (Fig. 4). Level-specific analyses further indicated that transport injuries were more strongly associated with thoracic than lumbar involvement (Fig. 4). The increased odds ratio in transport-related injuries compared to falls may be the result of the population we evaluated in the study. Texas is the second-largest U.S. State and is larger than any country in Europe. Given the wide geographical footprint, individuals from Texas are more likely to engage with motor vehicle transportation, particularly in rural areas where public transportation is not available. In addition, reduced mobility during lockdowns likely lowered short-term fall risk; however, prolonged inactivity and delayed healthcare may have contributed to a sharp rise in fall-related tSCIs after 2020 (16, 20-22).

We also found that assault- and homicide-related SCIs increased for thoracic and lumbar injuries during the study period (Fig. 2A, 4), consistent with broader trends in interpersonal violence observed following large-scale societal disruptions. This sustained rise aligns with prior evidence suggesting that domestic violence often increases in the aftermath of large-scale crises (23), influenced by heightened stress, social isolation, and disruptions in essential services. In addition, pandemic-related lockdowns likely intensified risk by forcing victims into prolonged contact with abusers while restricting access to support systems and external oversight. Given the vulnerability of populations most at risk for abuse-related SCI, particularly children, these trends underscore the importance of strengthening social safety networks and maintaining accessible reporting mechanisms during public health crises.

Fire-, burn-, and firearm-related tSCIs increased after the pandemic period, with the clearest rise occurring following the pandemic period, consistent with reports showing post-pandemic growth in both violent and accidental firearm injuries (24, 25). Although fire-, burn-, and firearm-related injuries contributed a smaller proportion of overall SCI risk compared with falls, they were disproportionately associated with thoracic-level injuries, indicating a distinct anatomic injury profile. Prior research has identified the home as the most frequent location for unintentional firearm injuries (26).

### Vertebral Regions

Spinal cord injury distribution by anatomical region demonstrated clear temporal and mechanistic patterns from 2019 to 2024. All spinal levels exhibited substantial declines during the pandemic, followed by a sharp rebound. This rebound paralleled the broader post-pandemic increase in overall SCI incidence and reflected a resurgence across both single-level and multi-level injuries. Our data support previous work that has found that the cervical spinal cord is most vulnerable to trauma due to least protection from the vertebral column (Fig. 2A) (27). Overall, our results suggest that cervical tSCI remain most common in transport injuries, while thoracic and lumbar are more associated with high-energy trauma and falls, respectively.

### Age, Sex, and Ethnicity

Adjusted analyses demonstrated substantial age-, sex-, and ethnicity-based disparities in SCI risk. Children and older adults consistently exhibited lower risk compared with young adults, whereas males and Black individuals experienced persistently higher risk across the study period (Fig. 2B, 4), findings consistent with prior studies (15). When considered alongside prior research showing that racial and ethnic minority individuals with SCI experience longer hospital stays and less favorable outcomes due to socioeconomic influences (28), these findings highlight the need for continued investigation into potential healthcare inequities. Given the persistent burden of tSCI among minority populations, future efforts should prioritize ensuring equitable access to prevention, treatment, and rehabilitation services. For example, future studies could evaluate relationships between tSCI location and rehabilitation services to estimate burden of medical care. In addition, more work is required to identify if rehabilitation approaches show similar efficacy among minority population.

### Public Health Region

The population analyzed in the present study allowed for a unique sub-analysis of geographic variation using public health regions (PHRs) (Fig. 3). In adjusted analyses, several regions—including PHRs 8 through 11—consistently demonstrated lower SCI risk relative to PHR 3 (Table 1), which encompasses the Dallas–Fort Worth metropolitan area. These regional differences likely reflect variation in population density, trauma system organization, transportation patterns, and access to specialized care.

Specifically, PHR 3 represents the largest population and ethnicity distributions (Table 1). But beyond the statistical associations, our data suggest that an individuals’ risk of having a tSCI is not necessarily dependent on location. For example, the overall odds of having a tSCI was similar in PHR 1, 2, 5 and 6 – locations ranging from large cities to rural areas. These findings highlight that tSCI may have substantial geographic variation. Prior literature (29, 30) has documented disparities in healthcare quality and disease burden across Texas, with poorer outcomes often observed in rural and underserved communities. Accordingly, future studies evaluating regional differences in healthcare access and quality may help identify targeted strategies to address the needs of specific populations. For example, individuals with tSCI in PHR 6 (Houston) may have access to more resources that will improve health outcomes compared to PHR 1 (Lubbock). In addition, our results suggest (Fig. 3) that specific areas of the region (e.g. PHR 2) may have a higher burden of disease management that could influence health outcomes.

### Limitations

This study has several limitations. First, the Texas trauma database is an administrative dataset that depends on accurate ICD-10 coding and reporting by participating hospitals. As such, misclassification of spinal cord injuries or causes of injury may have occurred. Second, the dataset includes only patients who presented to hospitals or trauma centers and received an ICD-10 diagnosis. Thus, our analysis excludes those who only received outpatient management or did not seek medical care, which may underestimate the true prevalence of SCI, particularly during the pandemic years when healthcare access was disrupted. Third, while data were available through 2024, reporting completeness and consistency may have varied across facilities and time, which may have influenced year-to-year comparisons. Additionally, some subcategories contained low case counts, meaning even minor numerical changes could appear statistically significant. Finally, the retrospective design limits causal inference, and the data does not capture long-term functional or rehabilitation outcomes. Despite these limitations, the study provides a comprehensive statewide overview of tSCI trends across a six-year period and identifies key demographic and mechanistic factors influencing injury risk before, during, and after the COVID-19 pandemic.

## Conclusion

This study demonstrates substantial temporal, mechanistic, and geographic variation in tSCI risk before, during, and after the COVID-19 pandemic in a population-based dataset. Adjusted analyses indicate that shifts in injury mechanisms, demographic risk profiles, and regional patterns persisted beyond the acute pandemic period, underscoring the dynamic nature of SCI risk during large-scale societal disruptions. These findings support the need for adaptable, mechanism-specific prevention strategies, strengthened injury surveillance, and regionally tailored interventions to mitigate SCI burden and enhance trauma system preparedness during and after public health crises.

## Figures and Tables

**Table 1:** Public Health Regions (PHR) in Texas. Data were based on recent public health reports (14).

Public Health Region (PHR)	2021 Population Estimates	Notable Cities	Hispanic (%)	White (%)	Black (%)	Asian (%)	Other (%)
**1 (Panhandle)**	~ 903,763	Amarillo, Lubbock	40%	50%	5%	2%	2%
**2 (North Central)**	~ 559,237	Wichita Falls, Abilene	25%	66%	6%	1%	2%
**3 (Dallas–Fort Worth)**	~ 8,226,141	Dallas, Fort Worth, Arlington	29%	46%	16%	7%	3%
**4 (Northeast TX)**	~ 1,163,913	Tyler, Longview	17%	65%	15%	1%	2%
**5 (Southeast TX)**	~ 786,778	Beaumont, Port Arthur	16%	60%	19%	2%	2%
**6 (Gulf Coast)**	~ 7,707,348	Houston, Pasadena	37%	35%	17%	8%	2%
**7 (Central TX)**	~ 3,662,025	Austin, Round Rock	30%	52%	10%	5%	3%
**8 (South Central)**	~ 3,190,195	San Antonio, New Braunfels	57%	33%	6%	2%	2%
**9 (West TX)**	~ 732,218	Midland, Odessa	54%	39%	4%	1%	2%
**10 (Far West TX)**	~ 905,742	El Paso	80%	14%	4%	1%	2%
**11 (South TX / Rio Grande Valley)**	~ 2,331,566	McAllen, Brownsville, Laredo	85%	13%	1%	1%	1%
